# Modeling the Morphometric Evolution of the Maize Shoot Apical Meristem

**DOI:** 10.3389/fpls.2016.01651

**Published:** 2016-11-04

**Authors:** Samuel Leiboff, Christopher K. DeAllie, Michael J. Scanlon

**Affiliations:** Plant Biology Section, School of Integrative Plant Science, Cornell University, IthacaNY, USA

**Keywords:** morphometrics, quantitative trait loci (QTL), discrete cosine transform, image analysis, *Zea*

## Abstract

The maize (*Zea mays* subsp. *mays* L.) shoot apical meristem (SAM) is a self-replenishing pool of stem cells that produces all above-ground plant tissues. Improvements in image acquisition and processing techniques have allowed high-throughput, quantitative genetic analyses of SAM morphology. As with other large-scale phenotyping efforts, meaningful descriptions of genetic architecture depend on the collection of relevant measures. In this study, we tested two quantitative image processing methods to describe SAM morphology within the genus *Zea*, represented by 33 wild relatives of maize and 841 lines from a domesticated maize by wild teosinte progenitor (MxT) backcross population, along with previously reported data from several hundred diverse maize inbred lines. Approximating the MxT SAM as a paraboloid derived eight parabolic estimators of SAM morphology that identified highly overlapping quantitative trait loci (QTL) on eight chromosomes, which implicated previously identified SAM morphology candidate genes along with new QTL for SAM morphological variation. Using a Fourier-transform related method of comprehensive shape analysis, we detected cryptic SAM shape variation that identified QTL on six chromosomes. We found that Fourier transform shape descriptors and parabolic estimation measures are highly correlated and identified similar QTL. Analysis of shoot apex contours from 73 anciently diverged plant taxa further suggested that parabolic shape may be a universal feature of plant SAMs, regardless of evolutionary clade. Future high-throughput examinations of SAM morphology may benefit from the ease of acquisition and phenotypic fidelity of modeling the SAM as a paraboloid.

## Introduction

The maize (*Zea mays* subsp. *mays* L.) shoot apical meristem (SAM) comprises a dome of pluripotent cells that ultimately generates all the organs of the plant shoot through regulated maintenance of stem cells and recruitment of initial cells for organogenesis (Steeves and Sussex, 1972). Mutational studies have shown that the maize shoot meristem morphology is genetically regulated (Jackson and Hake, 1999; Taguchi-Shiobara et al., 2001; Jia et al., 2009; Bommert et al., 2013; Pautler et al., 2015; Yang et al., 2015; Je et al., 2016). Although natural variation in shoot meristem morphology is associated with relatively few loci, natural variants of master regulatory genes do not appear to contribute to standing variation in SAM shape and size in domesticated maize (Thompson et al., 2014, 2015; Leiboff et al., 2015).

Recent investigations of maize meristem morphology as a quantitative trait incorporated small numbers of descriptive measurements approximating SAM shape and size for genome-wide association studies (GWAS) and quantitative trait locus (QTL) mapping (Thompson et al., 2014, 2015; Leiboff et al., 2015). Quantitative morphological analyses are highly biased by the measurement methodologies, the traits selected for analyses, and correlations between measurements (Langlade et al., 2005). Our previous study of maize inbred varieties exploited similarities between observed SAM contours and parabolic functions to estimate several shape parameters describing meristem morphology (Leiboff et al., 2015), although other models for SAM morphometrics have not been tested in quantitative genetic analyses.

Progress toward the description of complex shapes utilizing Fourier transform methods has enabled unbiased interrogations of biological shape (Dommergues et al., 2007; Klingenberg, 2010). By processing carefully placed landmarks or object outlines, Fourier transform and related methods use multiple sinusoid harmonics to reproduce highly complex shapes (Claude, 2008). High-dimensional matrices of Fourier model parameters can then be separated by principle component (PC) analysis to identify subtle, often cryptic, variations in complex plant shapes (Chitwood et al., 2014). Previous studies characterizing leaf morphology in *Antirrhinum* spp. and *Solanum* spp. have utilized Fourier shape descriptors as quantitative traits in QTL analyses of evolutionary novelty (Langlade et al., 2005; Chitwood et al., 2013, 2014).

Collectively known as teosintes, the wild members of the genus *Zea* provide a rich, highly diverse genetic system for maize genomics (Doebley, 2004; Hufford et al., 2012; Hake and Ross-Ibarra, 2015). Crosses between *Zea mays* subsp. *mays* and its progenitor, *Zea mays* subsp. *parviglumis* have been used to understand the genetic basis for striking changes in plant morphology associated with the domestication of maize (Beadle, 1980; Doebley, 2004; Hung et al., 2012; Shannon, 2012; Huang et al., 2016). Although general morphology and ontogeny of inflorescence meristem (IM) development have been reported in the genus *Zea* (Sundberg and Orr, 1986, 1990; Orr and Sundberg, 1994, 2004), little is known about variation in vegetative SAM morphology outside of domesticated maize. To date no comparative study has described the morphospace, or collection of shapes for vegetative meristems within the genus *Zea.* Indeed, no putative genetic factors underlying differences in maize and teosinte SAMs have been proposed.

Beyond the genus *Zea*, nearly all land plants and some algal relatives have shoot meristem-like structures, defined by an obligate balance of stem cell maintenance and lateral organ production (Bierhorst, 1971; Steeves and Sussex, 1972; Evert and Esau, 2006). Whereas shoot meristem cellular anatomy is notably different between many plant clades (Bierhorst, 1971; Evert and Esau, 2006), recent transcriptomic and genetic characterization has uncovered both contrasts and similarities in shoot meristem regulation pathways between disparate clades and meristem anatomy types (Frank and Scanlon, 2015; Frank et al., 2015). It is currently unclear whether analogous shoot meristem structures share quantitative morphological characteristics.

This project utilizes a maize x teosinte (MxT) backcross population to examine the genetic architecture of SAM shape and size (Hung et al., 2012; Shannon, 2012; Huang et al., 2016). We show that complex shape descriptors generated by Fourier methods detect previously undescribed, but genetically attributable minor variations in meristem shape, although the majority of the genetic loci contributing to SAM shape that are identified by Fourier analyses overlap tightly with loci identified by modeling the SAM as a paraboloid. Testing this expectation with a broad sampling of plant taxa suggests that parabolic shape may be a universal feature of plant SAMs.

## Materials and Methods

### Plant Growth

Germplasm for all experiments was grown in 10 h-day conditions with 27°C daytime and 25°C nighttime temperatures in Percival A100 growth chambers (Percival Scientific, Perry, IA, USA). An intermediate day length was selected to promote maturation of genus *Zea* members (Emerson, 1924). Plants were watered up to twice daily. Kernels were planted with randomized positions within 98-well trays where all edge positions were filled with maize inbred B73. Soil media was a 1:1 mixture of Turface MVP (PROFILE Products LLC, Buffalo Grove, IL, USA) and LM111 (Lambert Peat Moss, Rivière-Ouelle, QC, Canada). Wild teosintes (Supplementary Data Sheet 1) were grown in 4 repeated experiments. MxT backcross lines (Supplementary Data Sheet 1) were grown in 2 repeated experiments. Plants were harvested 14 days after planting and quickly trimmed to approximately 1 cm × 1 cm × 0.5 cm SAM-containing tissue cassettes and fixed in FAA (3.7% formalin, 5% acetic acid, 50% ethanol in water) on ice, overnight.

### Histology and Image Acquisition

After overnight fixation in FAA, plant tissue was dehydrated through an ethanol dilution series, transferred to a 1:1 mix of ethanol and methyl salicylate, then transferred to methyl salicylate for clearing overnight. Fully cleared tissue was imaged by DIC with Nomarski optics on an Axio Imager.Z10 (Carl Zeiss Microscopy, LLC, Thornwood, NY, USA) with an AxioCam MRc5 camera. We captured near-median longitudinal optical sections using SAM apex contours and primordia appearance as morphological cues. To avoid the analysis of determinant, IMs in our study, we only processed images where the shoot apex displayed recent initiation of leaf primordia, a morphological marker of vegetative SAMs (Pautler et al., 2013). All MxT images were oriented so that the next primordium to initiate (P0) appeared on the left-hand side of the image.

Several shoot apex images of anciently diverged plant taxa were collected from high quality publications (Supplementary Data Sheet 1). Figures from printed texts were scanned at 300 dpi, 16-bit greyscale using an Epson Perfection 3490 photo scanner (Epson America, Long Beach, CA, USA).

A small number of shoot apex images from demonstrative plant taxa were collected from freshly fixed tissues (Supplementary Data Sheet 1). Shoot apical regions were harvested by hand from growing tissue, fixed overnight in FAA, and stained with a modified Feulgen method (as described in 10). After a brief destain, samples were dehydrated, cleared with methyl salicylate and imaged with a Leica TCS-SP5 confocal laser scanning microscope (Leica Microsystems Exton, PA, USA) using an argon ion laser (488 nm).

### Image Processing: Parabolic Estimation and Fourier Transform

Near-median DIC images were processed by custom ImageJ macros to extract meristem contours and measures of SAM height and SAM radius (as reported in 10). Using a custom Python script SAM height and radius were used to calculate a table of 8 parabolic estimators: height, radius, height to radius ratio (H/R), volume (Vol.), surface area (Surf. Area), arc length (Arc Len.), parabolic coefficient (Para. Coeff.), and cross-sectional area (Area) (as previously reported in 10).

Shoot apical meristem contours were digitized with an Intuos Draw Tablet (Wacom Technology Corporation, Portland, OR, USA) and used for both linear model fitting with the lm() function and Fourier transform with the Momocs package for R. All traced SAM coordinates were imported as open contours (data type Opn), thinned to 800 evenly spaced pseudo-landmarks, Procrustes aligned, and Fourier transformed by the discrete cosine transform in the Momocs package for R. Discrete cosine transform made use of 12 harmonics, the lowest number of harmonics that sufficiently recaptured variation.

### QTL Mapping

Using publically available genotype information for the MxT population from panzea.org, genotype and phenotype information were processed via the R/qtl package for R. MxT genotypes were coded as BC2S3 and mapped using the Kosambi algorithm. Single QTL were detected using the scanone() function. We used a 95% confidence threshold generated from 10,000 permutations to determine significant QTL. Bayesian 95% confidence QTL intervals were called using the bayesint() function to estimate QTL location.

### Statistical Analysis and Plotting

Descriptive statistical analysis, correlation analysis, Wilcoxon one-sided rank sum test, and two-way ANOVA were carried out using core R packages. Raw data were summarized according to replicate by BLUP + coefficient using the nmle package in R (Leiboff et al., 2015). All correlations report Pearson’s product-moment, r and were evaluated for statistical significance with the Fisher transformation. Maize inbred variety SAM shape and size data were collected from published datasets (Leiboff et al., 2015). Plots were produced using ggplot2 and R/qtl packages in R.

### Data Availability

Supplementary Data Sheet 1 contains the source and parabolic model fit information for anciently diverged plant apex images. Supplementary Data Sheet 2 contains SAM parabolic estimates from the genus *Zea*. Supplementary Data Sheet 3 contains SAM parabolic model estimates from MxT lines in unsummarized and BLUP+Coefficient form. Supplementary Data Sheet 4 details all significant QTL intervals as well as known GWAS candidate genes within those intervals. Supplementary Data Sheet 5 contains Fourier shape descriptor PCs in unsummarized and BLUP+Coefficient form.

## Results

### Diversity of Shoot Meristems in the Genus *Zea*

**FIGURE 1:**
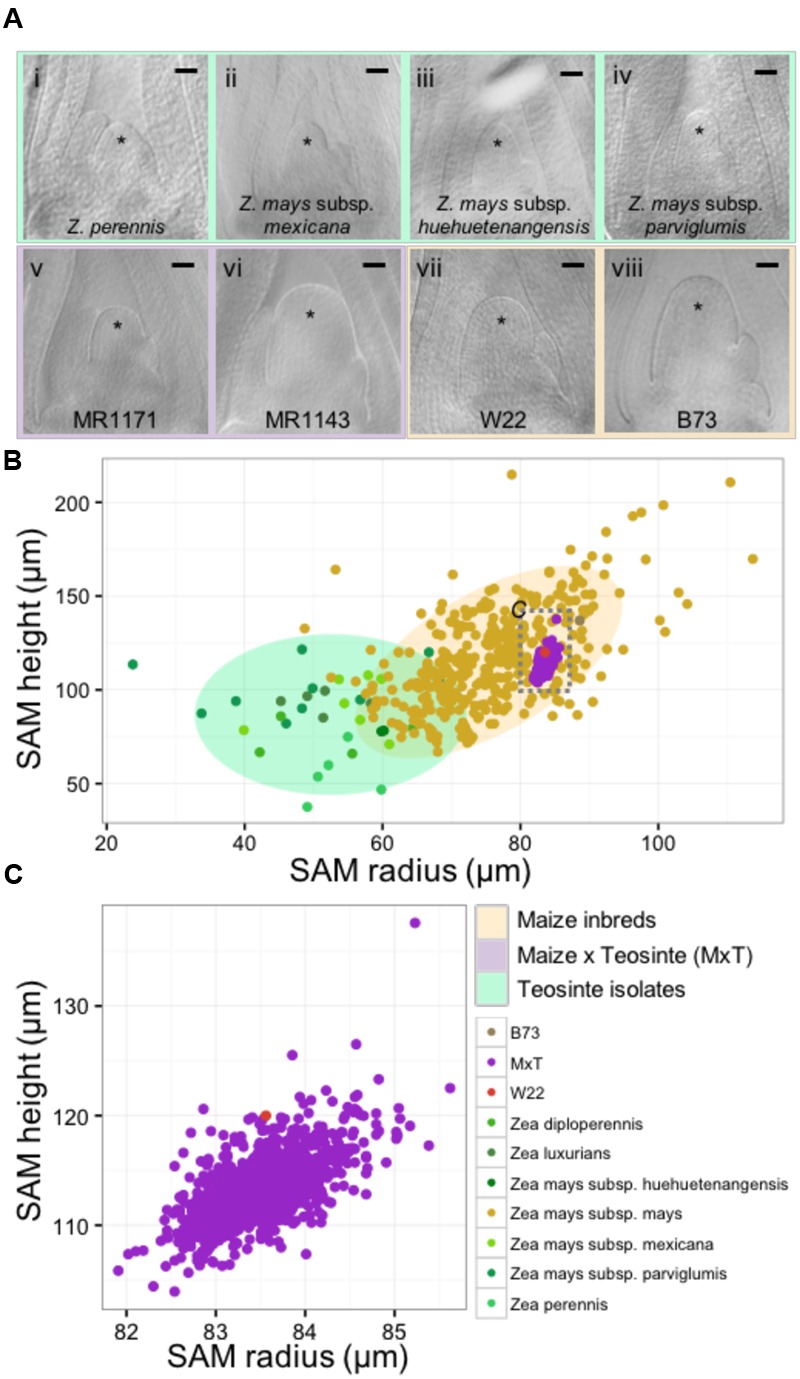
**The maize and teosinte shoot apical meristem (SAM) morphospace. (A)** Median longitudinal images of shoot meristems from wild teosinte isolates (i–iv), large and small SAMs from the maize x teosinte (MxT) intercross (BC2S3) lines (v–vi), and maize inbred varieties W22 (vii) and B73 (viii). ^∗^ indicates SAM with flanking leaf primorida. Scale bar, 50 urn. **(B)** Quantification of meristem radius and height show that SAM morphology observed in teosinte varieties (greens) and maize inbred varieties (yellow) partially overlap but teosinte SAMs are generally smaller than maize inbred SAMs. Measures from MxT lines (purple) cluster closely around the recurrent maize parent, inbred W22 (pink). Gray dashed box, location of data in panel **(C)**. Shaded ellipses, 90% density estimation of SAM shape data. **(C)** Variation in SAM shape and size in MxT lines (purple) around the W22 maize inbred parent (pink).

We utilized microscopic imaging of 14-day-old seedling vegetative SAMs (described in Materials and Methods) to construct a morphospace of SAM height and radius for the genus *Zea*, which included 33 wild teosinte isolates from 3 different species (*Z*. *diploperennis, Z*. *luxiurians*, and *Z*. *perennis*), 3 subspecies (*Z. may*s subsp. *huehuetenangensis, Z. mays* subsp. *mexicana*, and *Z. mays* subsp. *parviglumis*), 841 lines from a *Zea mays* subsp. *mays* W22 by *Zea mays* subsp. *parviglumis* backcross population (hereafter designated MxT) (Hung et al., 2012; Shannon, 2012; Huang et al., 2016), and our previously reported data on 369 diverse maize inbred lines (**Figure 1**; Supplementary Data Sheet 2) (Leiboff et al., 2015). Although there is a small zone of overlap between teosinte and maize inbred SAM shapes, wild teosinte meristems are significantly narrower (est. 23 μm between medians, Wilcoxon one-sided rank sum test, *p*-value <2.2*e-*16) and shorter (est. 28 μm between medians, Wilcoxon one-sided rank sum test, *p*-value = 1.257*e-*10) than meristems from domesticated maize inbred lines (**Figure 1A**). Measurements of MxT shoot meristems cluster around the recurrent maize parent, inbred W22 (**Figure 1B**), possibly reflecting the two generations of backcrosses to the maize parent that were incurred prior to analyses of SAM morphometric phenotypes (Hung et al., 2012). We detected quantitative variation in shoot meristem shape and size in SAMs isolated from MxT lines (**Figures 1A,C**) and focused our analysis on this population to understand the genetic architecture of maize/teosinte SAM morphometric variation.

### Parabolic Estimators of MxT Variation Identify New Meristem Morphology QTL

We used image-processing to collect two discrete measurements, SAM height and SAM radius and approximate the MxT shoot meristem as a paraboloid surface, the geometric shape yielded from revolving a parabolic curve around its central axis (additionally described in 10). Exploiting the simple geometry of a paraboloid, we used two primary measures to derive eight total parabolic shape estimators: height, radius, height to radius ratio (H/R), volume (Vol.), surface area (Surf. Area), arc length (Arc Len.), parabolic coefficient (Para. Coeff.), and cross-sectional area (Area) (Supplementary Data Sheet 3) (Leiboff et al., 2015).

**FIGURE 2:**
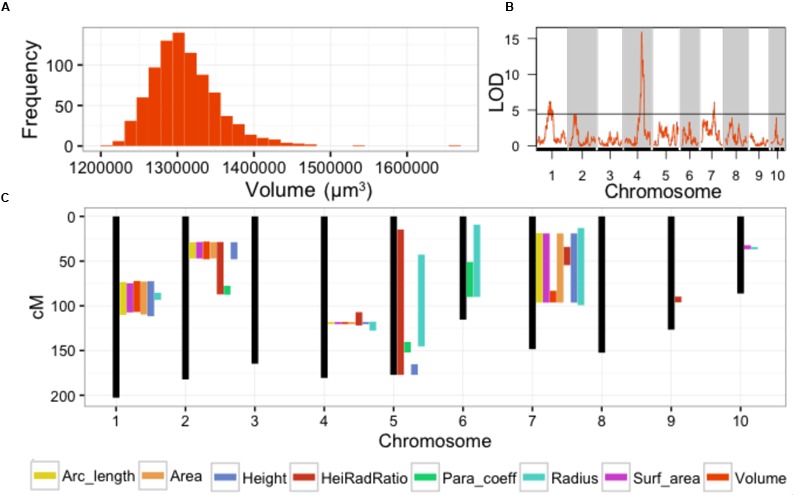
**Parabolic estimators of MxT line SAM shape and size identify meristem morphology **QTL. (A)** Volume measures of SAMs harvested from 841 BC2S3 MxT lines.** Measures are BLUP + coefficient values from 2 replicates. **(B)** Single QTL models detect significant loci for SAM volume on chromosome 1, 4, and 7. Black line, significance threshold *a* = 0.05. **(C)** Additional significant SAM morphology **QTL** are detected for all 8 parabolic estimators genome-wide.

Our previous study analyzed SAM volume (**Figure 2A**) as a quantitative trait (Leiboff et al., 2015). In this analysis we identified QTL for MxT SAM volume on chromosomes 1, 4, and 7 (**Figure 2B**). Intervals detected on chromosomes 4 and 7 were not previously implicated in natural variation of SAM morphology in 369 domesticated maize inbred varieties (Supplementary Data Sheet 4) (Leiboff et al., 2015). The large QTL interval identified on chromosome 1 contains several previously identified candidate genes for shoot meristem morphology including *ZmLAX*2, a putative auxin import protein which exhibits haplotype-specific differences in transcript accumulation patterns in maize inbred varieties that correlate with differences in SAM size (Leiboff et al., 2015).

The remaining 7 parabolic estimators mapped QTL to several chromosomes (**Figure 2C**; Supplementary Data Sheet 4). Several parabolic estimators identified highly overlapping QTL intervals. Chromosome 4, for example, contains a QTL that is coincidently associated with SAM height, radius, H/R, volume, surface area, arc length, and cross-sectional area (**Figure 2C**). All detected QTL were implicated by multiple parabolic estimators, except one QTL on chromosome 9 that is uniquely associated with SAM H/R. We find a high level of correlation between measures (Supplementary Image 1), as expected from their common derivation (see Materials and Methods).

In total, QTL intervals recaptured 11 previously identified SAM-morphology candidate genes implicated by GWAS of maize inbred varieties (Supplementary Data Sheet 4) (Leiboff et al., 2015). Intriguingly, the QTL intervals mapped on chromosomes 4, 7, and 9 have not previously been associated with SAM shape and size.

### Discrete Cosine Transform Uncovers Cryptic, Genetically Attributable Variation in MxT SAM Shape Variation

**FIGURE 3:**
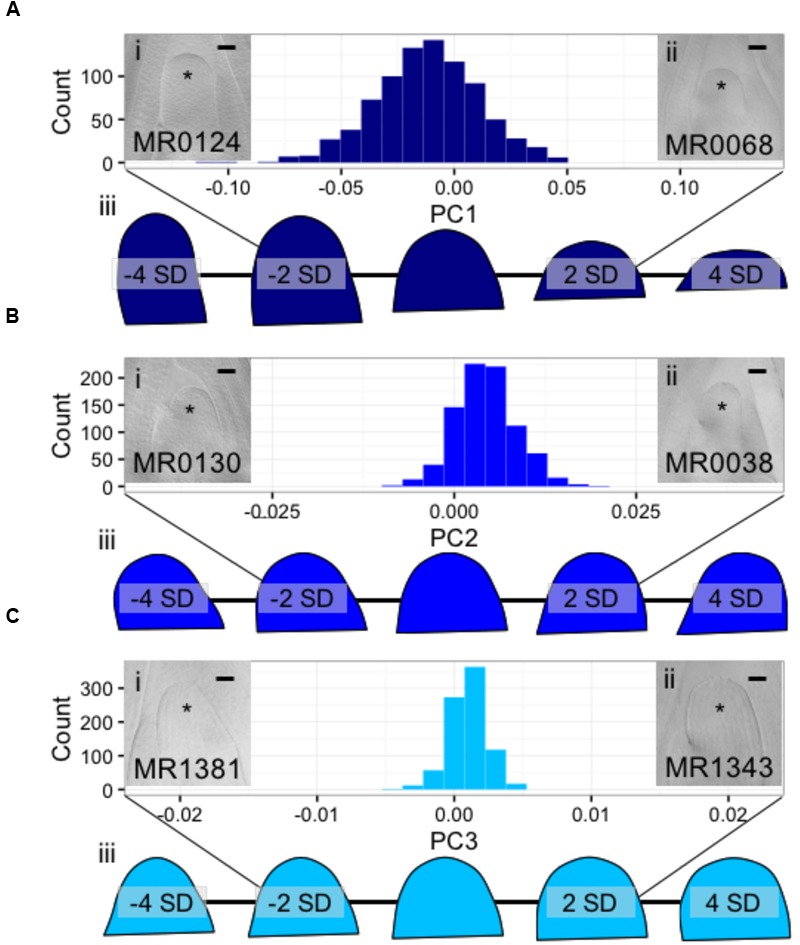
**Discreet cosine transformation identifies three SAM-morphology principal components (PCs) describing MxT meristem shape and size. (A)** PC1 identifies SAMs that vary from highly elongated, post-like to flattened, dome-like. **(B)** PC2 identifies asymmetrical SAMs that vary from left-leaning to right-leaning within the plane of histological sectioning. **(C)** PC3 identifies SAMs that vary from highly peaked to rounded. /’ and /’/, median longitudinal images of meristems from MxT lines with low and high values for each PC, respectively. ^∗^ indicates SAM with flanking leaf primorida. //’/, renderings of expected SAM shapes with PC values of -4, -2, 0, 2, and 4 standard deviations from the average recorded SAM shape. Histogram *x*-axes, -2 to 2 standard dev. Scale bars, 50pm.

Using Fourier- related transform methods, we processed Procrustes-aligned and scaled MxT shoot meristem outlines (Supplementary Image 2) into Fourier shape PCs to comprehensively describe SAM shape variation within this population (Supplementary Data Sheet 5). Three PCs describe more than 95% of the total observed shape variation, with PC1, PC2, and PC3, explaining 85.4, 8.2, and 2.3%, respectively. Additional PCs each explained less than 1% of observed shape variation (Supplementary Data Sheet 5); we therefore chose to focus our study of SAM shape variation on PC1, PC2, and PC3. Examining raw images of SAMs, and predicted shapes at the extremes of these PCs, revealed unexpected phenotypic variance (**Figure 3**). The majority of shape variation detected in the MxT population is attributed to PC1, identifying meristems that vary in appearance from ‘post-like’ to ‘dome-like’ (**Figure 3A**). PC2 identifies meristems that vary in 2-dimensional asymmetry with respect to the plane of sample dissection, and describes variation from ‘left-leaning’ to ‘right-leaning’ SAMs (**Figure 3B**). PC3 identifies meristems that vary in slope from the SAM base to tip, and includes ‘peaked’ to ‘rounded’ shapes (**Figure 3C**).

**FIGURE 4:**
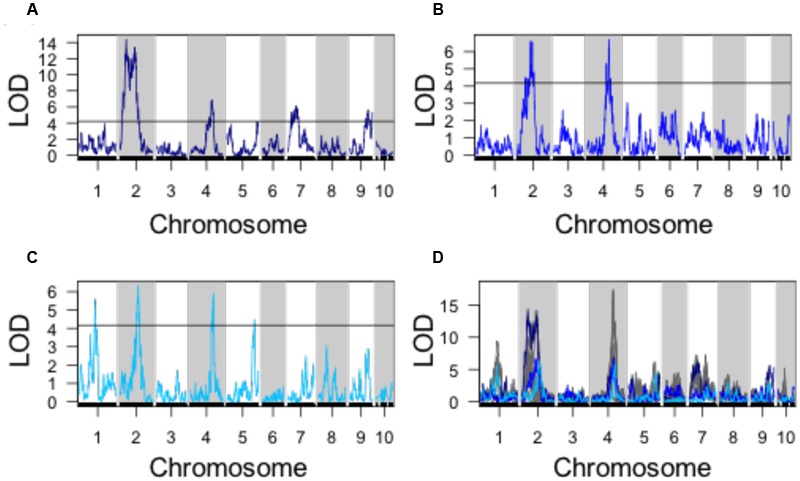
**QTL identified for discreet cosine transform PCs. (A)** QTL for PC1. **(B)** QTL **for** PC2. **(C)** QTL for PC3. **(D)** Superimposed QTL profiles for discreet cosine transform descriptors (blues) and parabolic estimator phenotypes (gray). Black line, significance threshold *a* = 0.05.

**FIGURE 5:**
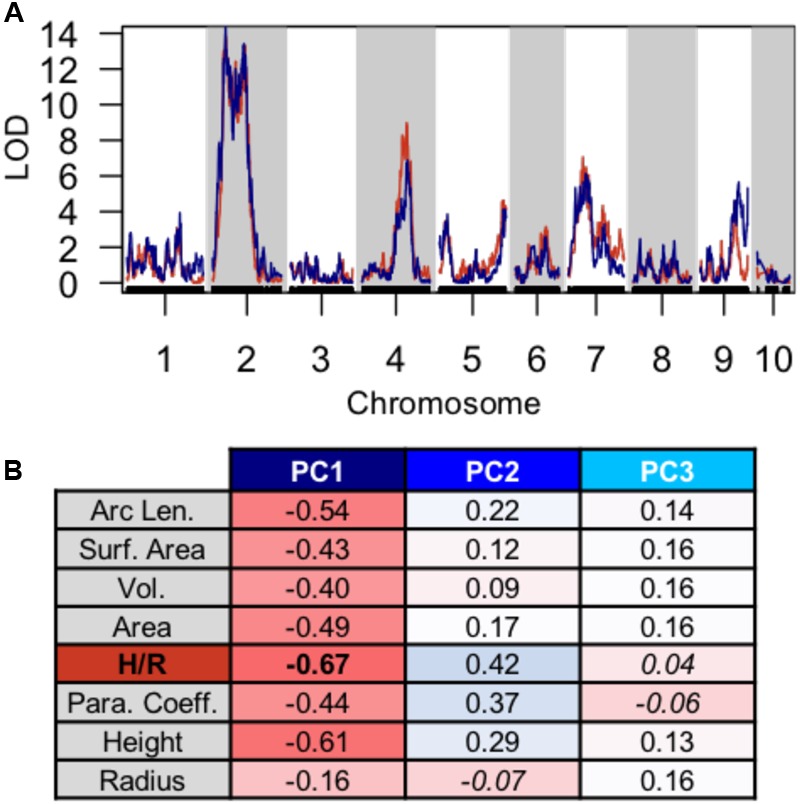
**Discreet cosine transform PC1 recaptures QTL and phenotypic variation identified by parabolic estimators. (A)** Close overlap of QTL identified using PC1 (blue) and H/R (red). **(B)** Strong pearson correlation (r) between PC1 and several parabolic estimator phenotypes. Correlation value, red to blue. Non-significant correlation Fischer transformation *P* > 0.05, italics.

Using PC1, PC2, and PC3 as quantitative phenotypes, we identified significant QTL for each trait: PC1 identified QTL on chromosomes 2, 4, 7, and 9 (**Figure 4A**), PC2 identified similar QTL intervals on chromosomes 2 and 4 (**Figure 4B**), whereas PC3 identified equivalent QTL intervals on chromosomes 2 and 4, in addition to different QTL on chromosomes 1 and 5 (**Figure 4C**). Despite differences in SAM measurement methods, the total QTL identified by all 8 parabolic estimators (**Figure 2C**) and 3 Fourier shape PCs overlap closely (**Figure 4D**). In a correlation analysis of parabolic estimators and Fourier shape PCs, we find a strong, significant correlation between PC1 and several parabolic estimators, especially SAM H/R (Pearson’s *r* = –0.67, Fisher transformation *p*-value < 2.2*e-*16) (**Figure 5A**). This close association is mirrored in the tight overlap of QTL identified by PC1 and H/R (**Figure 5B**). Because PC1 explains the majority of shape variation in the MxT population and is correlated in both numeric value and genetic architecture to parabolic estimators, we postulated that other populations of meristems might be likewise described by quantitative parabolic models.

### Diverse Meristems and Their Parabolic Models

**FIGURE 6:**
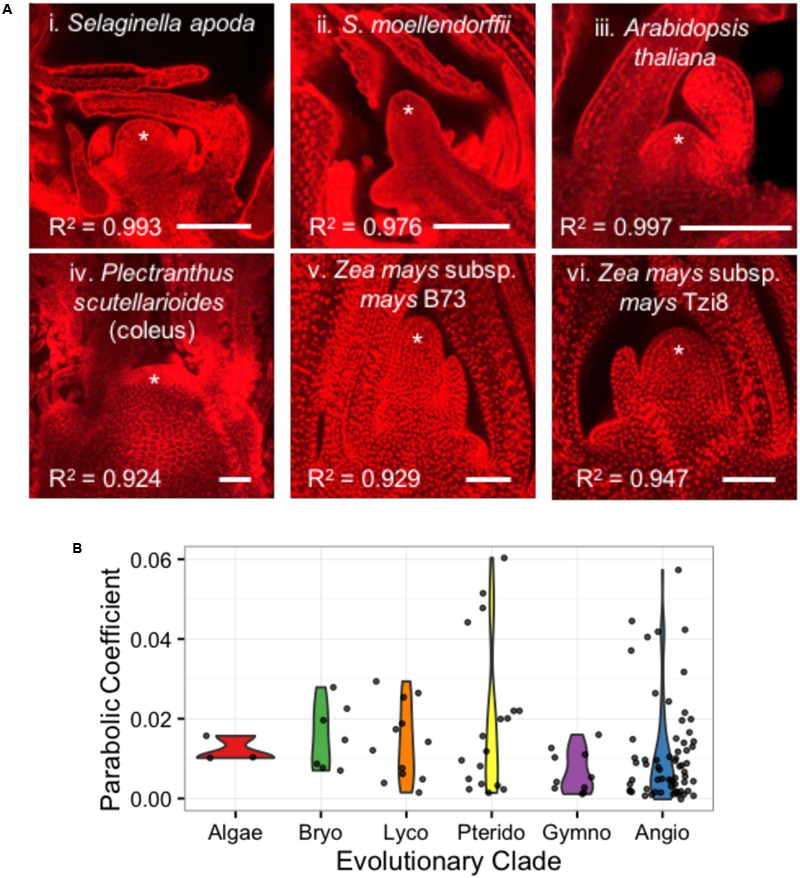
**Diverse SAMs are well-estimated by parabolic shape models. (A)** Median optical sections of the vegetative apex from example lycophytes with a shoot meristem dominated by an inverted pyramidal cell (i, ii) and angiosperms exhibiting a ‘tunica-corpus’ shoot meristem anatomy with at least one clonal ‘tunica’ layer (iii–vi). ^∗^ indicates SAM and flanking leaf primorida. R^2^, goodness-of-fit of parabolic model. Bar, 50 um. **(B)** SAMs from broadly diverse evolutionary lineages exhibit a range of parabolic coefficients.

Despite their similar roles in stem cell maintenance and the production of lateral organs, the shoot apices of anciently diverged plant lineages have remarkable anatomical and transcriptomic differences (**Figure 6A**) (Bierhorst, 1971; Evert, 2006a; Frank and Scanlon, 2015; Frank et al., 2015). In an analysis of 111 images from 73 plant taxa, we find that shoot apices are well fit by a parabolic model of meristem shape (Supplementary Data Sheet 1). Linear regression of shoot apex contours with a parabolic model yielded R^2^ goodness-of-fit scores ranging from 0.838 to 0.997 with a mean of 0.963 and median of 0.975. Interestingly, SAM shape parameters do not significantly separate anciently diverged evolutionary clades (ANOVA, *p*-value = 0.158) (**Figure 6B**).

## Discussion

We processed SAM images from 33 wild teosintes and 841 lines from a maize by teosinte (MxT) backcross population to generate a meristem morphospace for the genus *Zea*. In our SAM morphospace, we find that teosintes and maize inbred varieties occupy partially overlapping regions of the SAM shape, where SAMs in wild teosintes are diminutive compared to SAMs observed in domesticated maize varieties. We expect this SAM morphometric gradient to reflect the effect of domestication on the flowering time of *Zea mays*. The domestication and spread of maize outside its tropical Meso-American center of origin required adaptation to flowering during long summer days. Genetic studies of flowering time in maize, teosintes, and MxT intercross populations indicate that wild teosintes repress flowering during long days (Emerson, 1924; Briggs et al., 2007; Hung et al., 2012). Allelic variants which have decreased activity of *ZmCCT* (an ortholog of rice flowering time gene, *Gdh7*) allow flowering during long days and were selected for during the domestication of maize (Briggs et al., 2007; Tsuji et al., 2011; Hung et al., 2012; Yang et al., 2013). Previous studies of natural variation in maize inbred variety SAM shape and size revealed correlations between large meristem size and short flowering time (Leiboff et al., 2015; Thompson et al., 2015), reflected in GWAS candidate alleles at the *CONZ1* locus (Leiboff et al., 2015), proposed to act in a shared pathway with *ZmCCT* (Dong et al., 2012; Hung et al., 2012). QTL intervals for SAM surface area and radius on chromosome 10 include ZmCCT, which may contribute to differences in SAM size within the MxT population. Although the underlying mechanism that links flowering time and meristem size remains unresolved, our data agree with other reports of SAM natural variation and flowering time in maize.

Comprehensive analysis of SAM shape by Fourier methods yielded unexpected and interesting phenotypes for quantitative genetics. We note that the ‘left-leaning’ to ‘right-leaning’ asymmetry identified in PC2 (**Figure 3B**) only describes asymmetry in one plane of symmetry (i.e., the midrib-margin axis), and that additional asymmetric “leanings” may in fact exist along different axes in the shoot apex. We further note that PC2 variation may be attributed to physical crowding of the shoot apex, wherein genotype specific variations in the presence of leaf primordia that are closely appressed against the SAM may contribute to variations in SAM leaning. Likewise, the “peaked versus rounded” shape variation identified in PC3 may reflect differences in biophysical forces imparted by the youngest leaf primordium at the nodal disk-of-insertion at the base of SAM, wherein the base of the SAM is “pinched” at the node in “rounded” SAMs and “unpinched’ in peaked SAMs (**Figure 3C**). Prior implementations of Fourier methods for morphometrics have revealed genetically attributable, cryptic shape phenotypes including quantitative tissue asymmetry as we observed in PC2 (Langlade et al., 2005; Chitwood et al., 2013, 2014). Yet, because QTL identified with Fourier transform PCs overlap strongly with QTL identified by estimating the maize SAM as a paraboloid, we expect that parabolic estimation methods are effective at representing heritable variation in SAM morphology.

Approximating SAM shape and size with a parabolic model has several advantages for quantitative genetics. Parabolic estimates of SAM morphology can be generated by collecting two simple measures, SAM height and radius, whereas Fourier methods requires the careful placement of pseudo-landmarks or outline information generated from laborious manual image tracing or automated image processing of high signal-to-noise SAM micrographs. Despite the increased sensitivity of Fourier methods, we expect that the throughput of approximating SAM morphology with a paraboloid is better suited to large-scale genetic analyses of SAM morphology in maize.

We furthermore demonstrated that shoot apical regions from diverse plant taxa are well fit by parabolic curves. The shoot apices utilized in these comparisons (especially the historical samples) were obtained from plants grown in a wide variety of environmental conditions, which may indeed influence meristem size and/or geometry (Landrein et al., 2015). Our observations suggest that parabolic meristem shape is found in all plant evolutionary clades and SAM anatomical organization types (ex. tunica-corpus, histological zonation, single apical cell, etc.), which generally correlate with evolutionary clade. Interestingly, we found that anciently diverged plant lineages have similar shoot meristem parabolic curvatures, despite rich diversity in anatomy, development, and whole-plant morphology. The universality of parabolic SAM shape in diverse lineages may, in part, be the result of biophysical forces incurred during the essential functions of the SAM. All shoot meristems maintain at least one undifferentiated stem cell initial, which divides to produce both a stem cell initial and a lateral organ initial (Steeves and Sussex, 1972; Evert, 2006a,b,). In multicellular meristems, internal cellular division from the replication of initials places stress on epidermal cell walls, deforming the shoot apical domain into a parabolic shape (Niklas and Mauseth, 1980; Green, 1999; Kwiatkowska, 2004). Whereas the angiosperm tunica-corpus-type meristem comprises an outer, epidermal layer of cells under tension and at least one inner cell layer under compression, single apical cell-type meristems must accomplish similar geometries by portioning biophysical forces over just one cell wall and cytoplasm. Within possible parabolic shapes, our broad sampling of plant shoot meristems suggests that evolutionary clade alone is not a significant determinant of specific SAM parabolic shape; plant taxa from disparate evolutionary clades may have similar parabolic shapes. These data, therefore, constitute robust evidence for a fundamental biophysical model of meristem morphology, which transcends not only diverse evolutionary histories, but even an anatomical transition from single-celled structure to multicellular SAM.

As previous studies in maize have uncovered statistically significant correlations between SAM size and selected adult plant traits (Leiboff et al., 2015; Thompson et al., 2015), our analyses of SAM parabolic diversity within divergent plant taxa provide a framework for future investigations as to whether a fundamental correlation between SAM architecture and adult plant morphology may extend beyond phylogenetic boundaries.

## Author Contributions

MS, helped conceive and design the experiments, and edited the manuscript; SL, helped conceive and design the experiments, generated, analyzed, and interpreted the data, and wrote the manuscript. CD, performed imaging and image analyses of the teosinte SAMs.

## Conflict of Interest Statement

The authors declare that the research was conducted in the absence of any commercial or financial relationships that could be construed as a potential conflict of interest.
