# Supplementary figures and images for: Modeling the Morphometric Evolution of the Maize Shoot Apical Meristem
Source: Front Plant Sci. 2016 Nov 4;7:1651. doi: 10.3389/fpls.2016.01651 (PMC5095129; doi:10.3389/fpls.2016.01651)

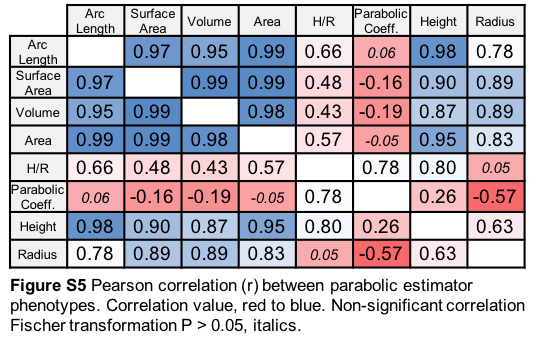

Supplement: Supplementary file 6 [file Image_1.png]

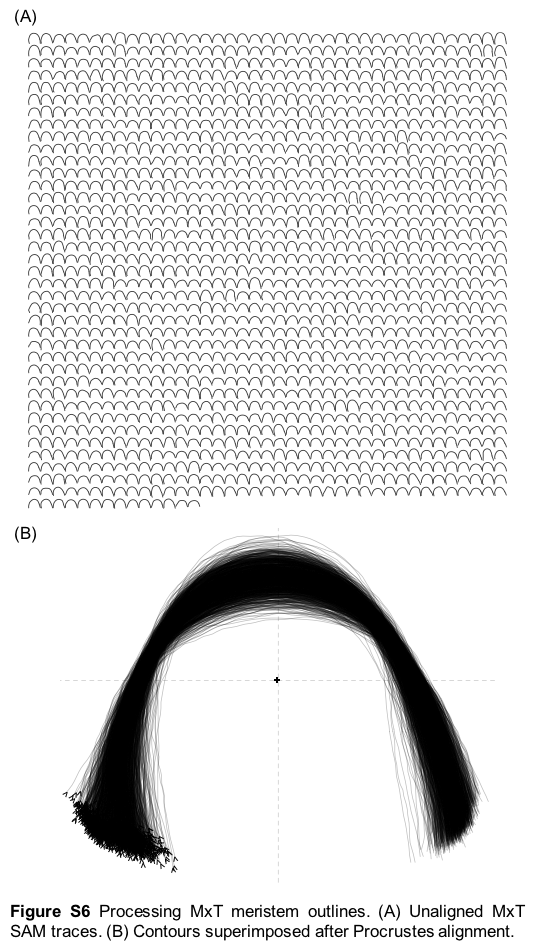

Supplement: Supplementary file 7 [file Image_2.png]
